# Smoking and alcohol use and healthcare professionals’ quality of life

**DOI:** 10.1192/j.eurpsy.2025.1005

**Published:** 2025-08-26

**Authors:** M. Κourakos, Z. Konstanti, A. L. Batiridou, C. Tsironis, K. Dimou, T. Mpakara-Nikou, T. Kafkia

**Affiliations:** 1 Department of Nursing, School of Health Sciences, University of Ioannina, Research Laboratory Psychology of Patients, Families & Health Professionals, Ioannina; 2 Department of Nursing, School of Health Sciences, International Hellenic University, Thessaloniki, Greece

## Abstract

**Introduction:**

Tobacco and alcohol use are two of the main risk factors for most of the non-communicable diseases and causes of death worldwide. Healthcare professionals can play an important role in helping patients to quit smoking and alcohol consumption, but this role is undermined if they themselves have the same addictions.

**Objectives:**

The aim of this study was to study the effect of smoking and alcohol use on healthcare professionals’ quality of life. In addition we aimed to identify any demographic and occupational factors that affect quality of life.

**Methods:**

The research sample of the present cross-sectional study consisted of 200 health professionals. The Demographic Questionnaire, the Smoking Addiction Inventory (Fagerstrom), the Alcohol Addiction Inventory, and the Quality of Life Inventory (EQ-5D) were used. The statistical analysis was performed using the statistical package IBM Spss v.27

**Results:**

Among the sample, the 46.5% stated that were smokers. The mean score of alcohol dependence was found to be 3.61 (±3.29), while the corresponding mean value of Fagerstrom was calculated to be 3.96 (±2.27). The EQ-5D quality of life score was found to be 0.93. Finally, the EQ-Vas score was found to be 7.95 (± 1.57).

**Conclusions:**

A particularly high prevalence of smoking was found among the healthcare professionals in the study, while alcohol consumption was moderate. The results showed that tobacco and alcohol consumption negatively affects the quality of life of healthcare professionals.

**Disclosure of Interest:**

None Declared

